# *FLOWERING LOCUS T* Triggers Early and Fertile Flowering in Glasshouse Cassava (*Manihot esculenta* Crantz)

**DOI:** 10.3390/plants6020022

**Published:** 2017-05-27

**Authors:** Simon E. Bull, Adrian Alder, Cristina Barsan, Mathias Kohler, Lars Hennig, Wilhelm Gruissem, Hervé Vanderschuren

**Affiliations:** 1 Plant Biotechnology, Department of Biology, ETH Zürich, 8092 Zürich, Switzerland; adrianalder@gmx.ch (A.A.); math.kohler@gmail.com (M.K.); wgruissem@ethz.ch (W.G.); 2 Gembloux Agro-Bio Tech, University of Liège, 5030 Gembloux, Belgium; cibarsan@ulg.ac.be; 3 Department of Plant Biology and Linnean Centre for Plant Biology, PO Box 7080, The Swedish University of Agricultural Sciences, SE-750 07 Uppsala, Sweden; Lars.Hennig@slu.se

**Keywords:** cassava, *Manihot esculenta* Crantz, flowering, *FLOWERING LOCUS T*, breeding, biotechnology, grafting, seed, recalcitrant crops

## Abstract

Accelerated breeding of plant species has the potential to help challenge environmental and biochemical cues to support global crop security. We demonstrate the over-expression of *Arabidopsis* *FLOWERING LOCUS T* in *Agrobacterium*-mediated transformed cassava (*Manihot esculenta* Crantz; cultivar 60444) to trigger early flowering in glasshouse-grown plants. An event seldom seen in a glasshouse environment, precocious flowering and mature inflorescence were obtained within 4–5 months from planting of stem cuttings. Manual pollination using pistillate and staminate flowers from clonal propagants gave rise to viable seeds that germinated into morphologically typical progeny. This strategy comes at a time when accelerated crop breeding is of increasing importance to complement progressive genome editing techniques.

## 1. Introduction

Rapid improvement and commercialization of woody perennial plant species is often hampered by lengthy breeding cycles [1]. This obstacle has gained prominence in recent years with the widespread uptake of genome editing techniques, notably CRISPR-Cas9 [2] and the need to segregate out T-DNA after site-specific editing of the genome [3,4]. Genome editing is revolutionizing agricultural breeding, but for many crops including cassava (*Manihot esculenta* Crantz), lengthy life cycles and limited fertility are major bottlenecks in harnessing this technology. Cassava is a prime candidate for accelerated breeding and genomic selection [5] because the starch-rich storage roots are both a staple food, particularly in sub-Saharan Africa, and a multi-billion dollar commodity in countries such as Brazil, China, and Thailand [6,7].

In cassava, time to flowering remains highly dependent on genotype and environmental conditions. Many farmer-preferred cultivars are non-branching to facilitate farming practices and to maximize stem growth for subsequent vegetative propagation. However, erect architecture is also associated with poor flowering capacity, with many cassava cultivars taking more than nine months to establish flowers in the field [8,9] and almost never flower under glasshouse conditions. In vitro manipulation of cytokinins have induced flowering in the laboratory [10] but controlled induction of stable inflorescence and seed production has so far remained elusive. The inefficacy of seed production is exacerbated by asynchronous flowering time, whereby monoecious pistillate flowers open one to two weeks prior to staminate flowers [11,12]. This gives rise to a highly heterozygous gene pool and complicates breeding such that introgression of desirable traits can take up to 15 years [9,13]. Overcoming poor seed production has been the focus in the development of alternative technologies, including synthetic seeds, permitting rapid multiplication and dissemination of cassava cultivars [14].

Flowering is a highly complex developmental process but the identification of FLOWERING LOCUS T (FT) [15] has prompted manipulation for advanced breeding initiatives. FT is a small globular protein produced in phloem companion cells where it interacts with FT-INTERACTING PROTEIN1 for movement to the sieve elements. Once in the phloem, FT is translocated to the shoot apical meristem where interaction with the bZIP transcription factor FD and phospholipid phosphatidylcholine [16] results in nuclear localization and activation of *LEAFY* (*LFY*), *APETALA1* (*AP1*), and *SUPPRESSOR OF OVEREXPRESSION OF CONSTANS1* (*SOC1*) to trigger flower development [17,18,19]. With improved understanding of flowering mechanisms, over-expression of *FT* has been exploited to induce precocious flowering in various plant species [20,21,22,23,24,25] thus enabling a more rapid and refined approach to breeding.

The expeditious advancement of genome editing technology, e.g., CRISPR-Cas9, has elicited a revival of tissue culture studies and rapid breeding strategies to enable segregation of editing tools in crop development [3,26]. Here we demonstrate the capacity to induce early flowering in glasshouse-cultivated cassava via the over-expression of *Arabidopsis FT* and, importantly, to enable sexual reproduction, yielding viable seeds that germinate into healthy progeny.

## 2. Results

**Figure 1:**
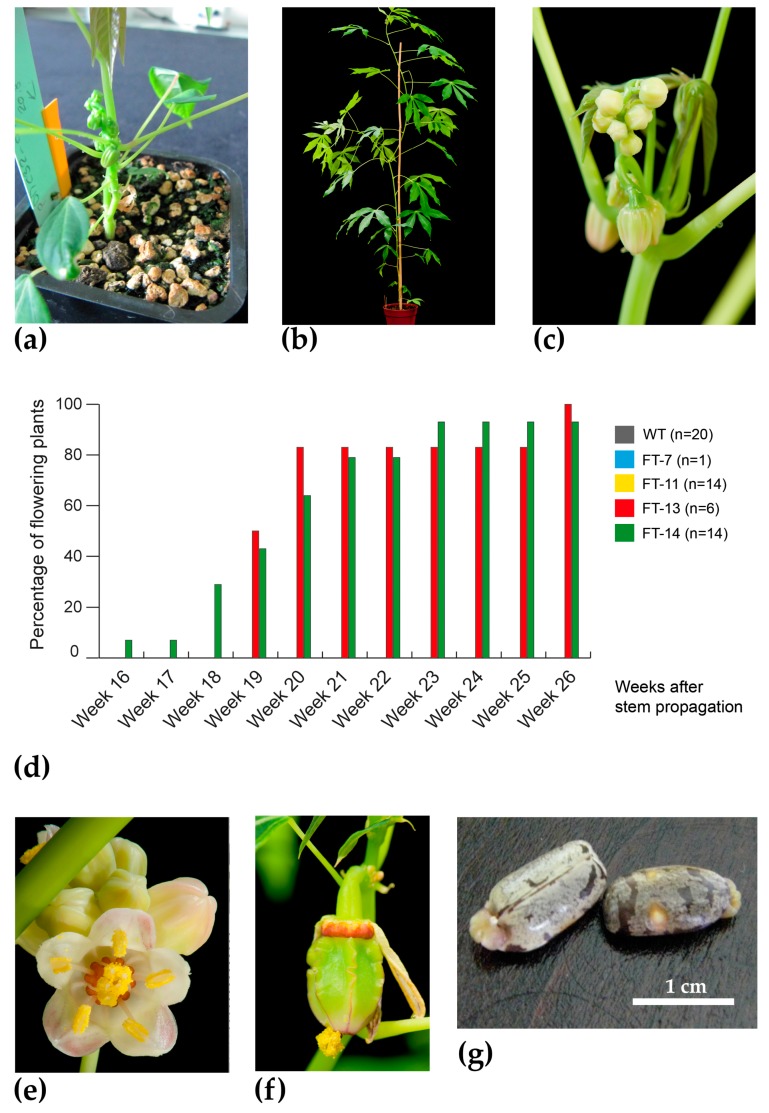
Early flowering in glasshouse-cultivated cassava. (**a**) FT-14 plant with flower buds evident after six weeks of growth in soil; (**b**) FT-13 plant at five months of growth; and (**c**) enlarged image showing pistillate and staminate buds on a branch; (**d**) Percentage of flowering plants per line following stem propagation; (**e**) Staminate flower from transgenic line FT-13; (**f**) Seed capsule developing after manual pollination of a pistillate flower with pollen of transgenic line FT-13 (selfing); (**g**) Harvested cassava seed resulting from manual pollination. Glasshouse conditions were 26 °C, 60% humidity and 14 h photoperiod with supplementary lighting.

A binary expression cassette comprising the coding sequence of *AtFT* constitutively expressed by a *CaMV35S* promoter was assembled. Putative transgenic in vitro plantlets derived from *Agrobacterium*-mediated transformation of the model cultivar 60444 were screened for the presence of the *CaMV35S*:*AtFT* construct using a rooting assay, PCR amplification of the selection marker *hptII*, the *AtFT* transgene (Figure S1), and Southern blot analysis using a DIG-labelled probe to *hptII* (Figure S2). RT-PCR of selected plantlets revealed transgene expression in lines FT-11, FT-13, and FT-14 (Figure S3). These lines, a control (line FT-7, which contains T-DNA with *hptII* but lacking *AtFT*), and wild-type cultivar 60444 (60444 WT) were propagated in vitro and six or seven plants per line were transferred to soil in the glasshouse. After only six weeks, flower development was observed in two plants of line FT-14 (Figure 1a), although these flowers did not develop to maturity. Approximately 15 weeks under glasshouse cultivation, all six of the FT-13 plants and six of the seven FT-14 plants had floral buds or flowers. Flowering was predominantly at the apical growing region but a branched phenotype with terminal inflorescence and typical arrangement and number of pistillate and staminate flowers was observed (Figure 1b,c). No flowers developed in any of the seven plants of line FT-11 but this observation was not investigated further. As expected, no flowers were observed in any of the FT-7 and 60444 WT plants. Due to the asynchronous flowering of plants of lines FT-13 and FT-14, it was not possible to manually pollinate pistillate flowers.

Due to the asynchronous flowering of cassava, stems made from the mature plants were vegetatively propagated to increase plant number and improve the likelihood of concurrent fertile cyathia production to allow pollination studies. Flower buds were first observed in a plant of FT-14 at 16 weeks after planting (Figure 1d), yielding morphologically normal flowers (Figure 1e). Flowering peaked in weeks 18–20 post propagation when 13 plants from lines FT-13 and FT-14 flowered (Figure 1d). In-keeping with results from the previous experiment, no plants of lines FT-7 and FT-11 produced flowers. Manual pollination (Figure S4) of pistillate flowers of FT-13 was performed successfully during a six-month period. Dehiscence of the capsules released two seeds from each crossing after approximately 11 weeks of maturation (Figure 1f,g), with the exception of the final cross in which three seed were formed. In total, 12 from 13 seeds germinated to yield morphologically normal cassava; the exception was a seed from the three-seed capsule that failed to germinate and was presumed aborted.

To investigate whether further vegetative propagation of the *AtFT* expressing transgenic lines affected flowering capacity, multiplication of stems of FT-13 (14 plants), FT-14 (18 plants), and 60444 WT (47 plants) was performed. As observed previously, inflorescence initiation was noted after only six weeks of growth with flowers forming at the apical growing region. After approximately 12 weeks, 64% of FT-13 plants and 33% of FT-14 plants had developing flowers. None of the 47 wild-type plants produced flower bud primordia. This data suggests vegetative propagation of the transgenic material does not alter the capacity of the *AtFT* transgene to induce flowering.

With the successful accelerated flowering in the transgenic lines FT-13 and FT-14, we tested if grafting of a non-flowering 60444 WT scion on the rootstock of an FT line could induce flowering. 60444 WT scions were grafted to FT-13, FT-14, or 60444 WT control rootstocks (3, 7, and 10 plants per line, respectively) and maintained under glasshouse conditions. However, even after 10-months growth, none of the grafted plants flowered. It is not clear why flowering was halted, but it may be associated with an inability of rootstocks to generate sufficient amounts of mobile FT, which is normally loaded into the phloem in leaves. This notion is consistent with the observation that transgenic rootstocks retained a limited (approximately one to three) number of leaves under glasshouse conditions due to natural leaf shedding observed in cassava over a 10-month period. Additionally, rootstocks with a single stem were chosen to improve grafting success.

## 3. Discussion

Our results demonstrate the capacity of stably transformed cassava cultivar 60444 expressing *AtFT* to initiate flower development in glasshouse conditions. Moreover, propagation of plants to maturity allows fertile flowers to be crossed and viable seed to be harvested within one year. To our knowledge, this is the first report of accelerated flowering and viable seed production in cassava using *Arabidopsis FT*. This study complements other reports of induced flowering [20,21,22,23,24,25,27], further highlighting the importance to continually develop efficient methods to foster flowering in cassava and other important crops. Similar to our grafting experiment, a recent field study also revealed a lack of flowering in non- or late-flowering cultivars grafted to rootstocks of a profuse, early flowering (non-transgenic) cultivar [28]. Whilst progress has been made, it is apparent further studies are required to improve our understanding of floral signals [29] in this important crop.

To date, advances in conventional breeding and biotechnology have been slowed by the poor flowering capacity of many farmer-preferred cultivars and exacerbated by heterogeneity, leading to lengthy breeding programs to introgress desirable traits. Successful induction of flowering in cassava would help maximize breeding programs [30,31], not only in tropical regions but also for crossing of varieties in environments where flowering is seldom seen, namely, glasshouses in temperate countries. Cassava breeding programs have sourced improved traits from wild relatives, including resistance traits against viral pathogens [14,32]. We anticipate that the proof-of-concept *AtFT* expression in cassava will also become instrumental to either facilitate introgression of improved traits or accelerate domestication of wild *Manihot* species. For example, there is tremendous scope to utilize CRISPR-Cas9 for genome editing of domestication genes in parallel to accelerated flowering lines, the progeny of which can be screened for null mutants for the transgene but which have improved traits [33]. The development of crop varieties using these new techniques offers an opportunity to improve agricultural diversity and sustainability.

## 4. Materials and Methods

### 4.1. Expression Vector Assembly and Bacterial Transformation

*Arabidopsis FLOWERING LOCUS T* (*AtFT*; AT1G65480) coding sequence was cloned into the pENTR™/D Gateway™ vector (Invitrogen, Carlsbad, CA, United States) and then into the pMDC32 Cassette 1 (pMDC32-C1) destination vector [34] via an LR reaction (Invitrogen guidelines). *AtFT* expression was controlled by the proximal enhanced *Cauliflower mosaic virus* (*CaMV*) 35S promoter and nopaline synthase (*nos*) transcription terminator sequence. Plant selection utilized the bacterial *hptII* (hygromycin B) resistance gene. The vector was transformed into chemically competent *Escherichia coli* (One Shot^®^ TOP10 competent cells; Invitrogen guidelines) and colonies were grown on LB agar plates containing 50 mg L^−1^ kanamycin antibiotic. Plasmid DNA was purified from a liquid culture (GeneJET, ThermoFisher Scientific, Waltham, MA, United States) and used to electroporate *Agrobacterium tumefaciens* strain LBA4404. Colonies were recovered on LB agar plates containing rifampicin 25 mg L^−1^, streptomycin 100 mg L^−1^ and 50 mg L^−1^ kanamycin. Intact plasmid (*pMDC32*-*AtFT*) uptake was verified by PCR amplification.

### 4.2. *Agrobacterium*-Mediated Cassava Transformation

Cassava cultivar 60444 (a Nigerian bred line) was transformed as described [35]. In brief, friable, embryogenic callus (FEC) derived from secondary somatic embryos was propagated on Gresshoff and Doy-based [36] medium (Duchefa Biochemie B.V., Haarlem, The Netherlands) supplemented with the synthetic auxin, picloram (12 mg L^−1^). Two independent batches of FEC generated from cassava cultivar 60444 were used for transformation. *Agrobacterium* harboring the *pMDC32-AtFT* plasmid (see above) were cultured in LB until growth reached optimal density. Resuspended bacteria were co-cultivated with the FEC on solid medium prior to selection and regeneration via embryogenesis [35].

### 4.3. Cassava Regeneration and Glasshouse Cultivation

Developing cotyledonary embryos were transferred to Murashige and Skoog (MS)-based [37] medium (Duchefa Biochemie B.V., Haarlem, The Netherlands) supplemented with 6-Benzylaminopurine (a synthetic cytokinin) to promote plant growth and shoot development. Apical growing tips were isolated and planted in MS medium containing 10 mg L^−1^ hygromycin B for selection; plantlets were screened after two weeks and only those that had developed roots were maintained for further analysis. In vitro plantlets were propagated via stem sections and cultured for four weeks (28 °C, 16 h photoperiod) prior to transfer to soil pots maintained in the glasshouse [35].

### 4.4. PCR Amplification to Screen Putative Transgenic Plantlets

Genomic DNA was extracted from in vitro leaf samples frozen in liquid nitrogen using a modified protocol [38] and used as template material in PCR. Reactions contained 1X DreamTaq buffer, 0.2 mM dNTPs, 1 µM SuperFT-F primer (5′-AGACCCTCTTATAGTAAGCAGAG-3′), 1 µM SuperFT-R primer (5′-TACACTGTTTGCCTGCCAAG-3′), sterile, distilled water, 200 ng genomic DNA, 2.5 U DreamTaq DNA Polymerase (ThermoFisher Scientific, Waltham, MA, United States). Reactions were cycled with an initial step 95 °C (1 min), then 35 cycles of 95 °C (1 min), 57 °C (1 min), 72 °C (1 min), then a final step of 72 °C (5 min). The products were resolved in a 1% TAE agarose electrophoresis gel containing ethidium bromide and visualized alongside a 1 Kb molecular marker (GeneRuler™).

### 4.5. Southern Blot Analysis

Genomic DNA was extracted from in vitro leaf samples frozen in liquid nitrogen using a modified protocol [38]. Quality and quantity of DNA samples were determined by NanoDrop™ (ThermoFisher Scientific, Waltham, MA, United States). 10 µg DNA was restriction enzyme digested (*Hind*III; New England Biolabs, Ipswich, MA, United States) for 16 h and subsequently ethanol precipitated and resuspended in 20 µL sterile, nuclease-free water prior to loading on a 1% TAE agarose gel, including a DIG-labeled marker (Roche, Basel, Switzerland). DNA was transferred to nylon membrane via Southern blotting [39] and hybridized with a DIG-labeled probe targeting *hptII*. T-DNA integration events were determined following exposure to autoradiograph film.

### 4.6. RT-PCR for AtFT Transgene Expression

Total RNA was extracted from in vitro leaf samples frozen in liquid nitrogen using a modified protocol [38]. Quality and quantity of RNA samples were determined by NanoDrop™ (ThermoFisher Scientific, Waltham, MA, United States) and subsequently validated in a 2% agarose electrophoresis gel. Samples were treated with DNase according to the manufacturer’s guidelines (RQ1 RNase-Free DNase; Promega, Madison, WI, United States) and cDNA prepared using the RevertAid First Strand cDNA Synthesis Kit and random hexamer primer (ThermoFisher Scientific, Waltham, MA, United States). RT-PCR contained 1X DreamTaq buffer, 0.2 mM dNTPs, 1 µM SuperFT-F primer (5′-AGACCCTCTTATAGTAAGCAGAG-3′), 1 µM LH286-R primer (5′-CTAAAGTCTTCTTCCTCCGCA-3′), sterile, distilled water, 1 µL cDNA, 2.5 U DreamTaq DNA Polymerase (ThermoFisher Scientific, Waltham, MA, United States). Amplification of the *PP2A* reference sequence used oligonucleotides PP2A-F (5′-TGTGGAAATATGGCATCAATTTTGG-3’) and PP2A-R (5’-GCAACAGAAAGCCGTGTCAC-3′). Reactions were cycled with an initial step 95 °C (1 min), then 35 cycles of 95 °C (30 s), 60 °C (*AtFT* primers) and 58 °C (*PP2A* primers) (30 s), 72 °C (15 s), then a final step of 72 °C (10 min). The products were resolved in a 1% (*AtFT* amplification products) or 2% (*PP2A* amplification products) TAE agarose electrophoresis gel containing ethidium bromide and visualized alongside a molecular marker (GeneRuler™).

### 4.7. Flowering, Pollination, and Seed Germination

In vitro plantlets and stem cuttings of each selected transgenic line and wild-type 60444, were grown in the glasshouse at 26 °C, 60% humidity and 14 h photoperiod with supplementary lighting. Upon flowering, staminate flowers were removed and used to pollinate the pistillate flowers (from a clonal propagant). After 10–12 weeks, seed was harvested and germinated on soil in the glasshouse. Plants were watered daily and fertilized (Wuxal^®^) fortnightly.

### 4.8. Grafting of Cassava Stems

Cassava scions were cleft grafted under glasshouse conditions following a modified procedure [40] and described previously for cassava [41]. Cassava scions with at least two internodes were grafted onto rootstocks with similar stem diameters. Grafts were joined using Parafilm^®^ and the apical part of each scion was coated with wax.

## Supplementary Materials

The following are available online at www.mdpi.com/2223-7747/6/2/22/s1, Figure S1: PCR-amplification products using *pMDC32-AtFT* putative transformed in vitro plantlets; Figure S2: Southern blot hybridization of *Hind*III digested genomic DNA from selected in vitro plantlets transformed with *pMDC32-AtFT*; Figure S3: RT-PCR performed on selected *pMDC32-AtFT* lines; Figure S4: Manual pollination of a pistillate flower from a glasshouse-cultivated transgenic FT-13 plant.
