# Supplementary material for: FLOWERING LOCUS T Triggers Early and Fertile Flowering in Glasshouse Cassava (Manihot esculenta Crantz)
Source: Plants (Basel). 2017 May 27;6(2):22. doi: 10.3390/plants6020022 (PMC5489794; doi:10.3390/plants6020022)
Supplement: Supplementary file 1 [file plants-06-00022-s001.pdf]

Supplemental Materials: *FLOWERING LOCUS T* Triggers Early and Fertile Flowering in Glasshouse Cassava (*Manihot esculenta* Crantz)

Simon E. Bull, Adrian Alder, Cristina Barsan, Mathias Kohler, Lars Hennig, Wilhelm Gruissem and Hervé Vanderschuren

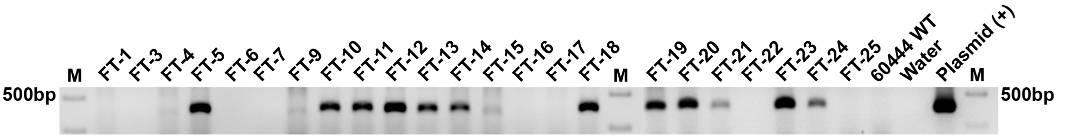

Figure S1. PCR-amplification products using *pMDC32-AtFT* putative transformed in vitro plantlets. Reactions contained oligonucleotide primers specific to the *AtFT* transgene. Products from individual plants (FT-1 to FT-25); 60444 WT, negative control; Water, PCR water control; Plasmid (+), *pMDC32-AtFT* positive control. bp size markers (lanes M). Products resolved in a 1% TAE agarose electrophoresis gel.

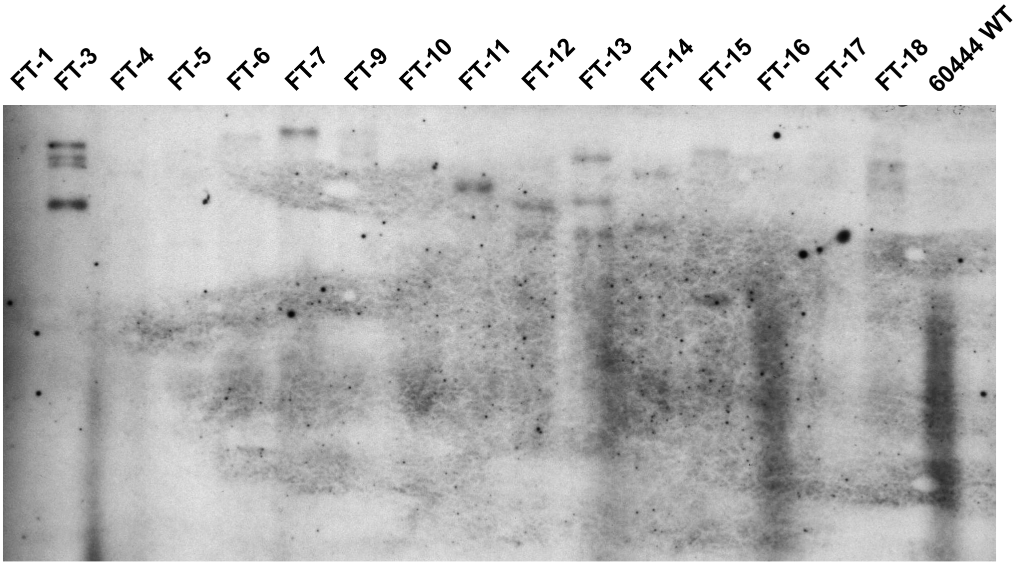

Figure S2. Southern blot hybridisation of *Hind*III digested genomic DNA from selected in vitro plantlets transformed with *pMDC32-AtFT*. Samples (FT-1 to FT-18) hybridised to a *hptII*-annealing DIG-labeled probe. 60444 WT, negative control. Lines FT-11 (one insertion event), FT-13 (three insertion events) and FT-14 (two insertion events) were selected for further analysis.

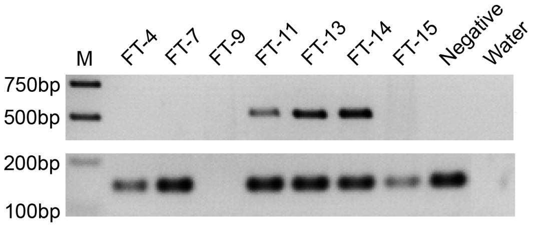

Figure S3. RT-PCR performed on selected *pMDC32-AtFT* lines. *AtFT* transcript detected only in lines FT-11, FT-13 and FT-14. Reactions using oligonucleotide primers specific to the *AtFT* transgene (upper image). Amplification control using endogeneous *PP2A* reference gene primers (lower image). Water, PCR water control; Negative, non-transgenic control plantlet; FT-7, vector control line. bp size markers (lane M). Products resolved in a 1 % (*AtFT* amplification products) or 2 % (*PP2A* amplification products) TAE agarose electrophoresis gel.

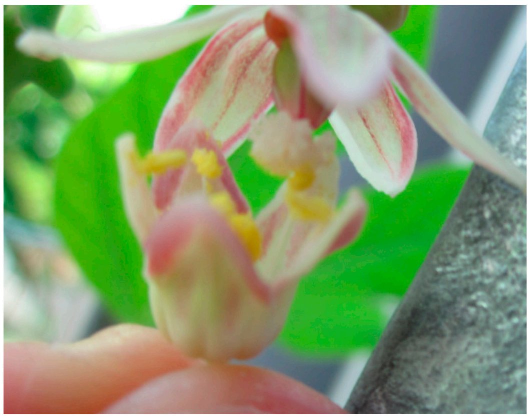

Figure S4. Manual pollination of a pistillate flower from a glasshouse-cultivated transgenic FT-13 plant.
